# Hydrophobia

**Published:** 1879-03

**Authors:** 


THE gardener of A. A. A., Esq., heard his neighbor of Fifth Avenue, say, on Tuesday, May 2[illegible], that his son had been bitten on the cheek by a dog, and that he would not have had it to occur for ten thousand dollars. This statement made such a strong impression on the gardener's mind as to the fearful nature of the bite of a dog, that, on reaching home, he complained of being unwell, and went to bed; in a short time he felt darting pains running up his arm towards the shoulder; subsequently, on offering him a drink of water, Mr. A. heard him make a noise like the bark of a dog, accompanied with a shuddering. The symptoms increased apace, and he died in horrible agonies on Friday, the fourth day.

Nine months before, this man was bitten on the complaining arm, but it soon healed, and nothing more was heard of it. This shows in a striking manner, what an influence the mind may have on the body. It is possible that the gardener might have had the attack, if he had not heard the conversation, but it is not probable, because he was in his usual good health.

We should learn from this not to nurse our symptoms, nor to allow the mind to dwell on any bodily infirmity, but if we have any ailment to make every possible effort to divert the current of thought, by mixing in cheerful society, engaging in good doing, or by embarking in some occupation which, while it secures bodily activity, compels the mind away pleasurably to things which feed and engross it. Earnest industry is a necessity of our nature; there is no persistent good health without it. No man was made to be a loafer. The idler, the do-nothing, is a drone among his fellows, the curse of his kind. All have much to do. The time is short, and the night cometh, when no man can work. Be busy, then, one and all.

**MUSH BISCUIT.**—Make about a quart of Indian-meal mush or stirabout; while hot, add a piece of butter, about the size of an egg, thin it with milk, adding a little salt; then add some flour, thin it with a tea-cup of yeast, then add as much more flour as will make it the consistence of dough; knead it well, set it to rise, and bake with a hot fire. The meal makes the bread light, and thus removes the objection to the unhealthfulness of hot bread.

